# Changes in Symptoms and Cognition Following Electroconvulsive Therapy in Late-Life Depression: Analyses Stratified by Baseline Anxiety Levels

**DOI:** 10.31083/AP51154

**Published:** 2026-09-09

**Authors:** Wenfeng Zhen, Han Wu, Shuo Lin, Dandi Zhu, Siyuan Lian, Li Ren, Yilang Tang, Yanping Ren, Xin Ma

**Affiliations:** ^1^Beijing Key Laboratory of Mental Disorders, National Clinical Research Center for Mental Disorders & National Center for Mental Disorders, Beijing Anding Hospital, Capital Medical University, 100088 Beijing, China; ^2^Advanced Innovation Center for Human Brain Protection, Capital Medical University, 100088 Beijing, China; ^3^Department of Psychiatry and Behavioral Sciences, Emory University School of Medicine, Atlanta, GA 30307, USA; ^4^Mental Health Service Line, Joseph Maxwell Cleland Atlanta VA Medical Center, Decatur, GA 30033, USA

**Keywords:** electroconvulsive therapy, late-life depression, anxiety, efficacy, cognitive function

## Abstract

**Background:**

Late-life depression (LLD) is commonly complicated by comorbid anxiety. However, whether the severity of baseline anxiety is associated with differential symptomatic and cognitive outcomes following electroconvulsive therapy (ECT) in LLD remains unclear, particularly with respect to cognitive safety in high-anxiety older patients.

**Methods:**

This prospective self-controlled pre–post study was conducted in accordance with the Strengthening the Reporting of Observational Studies in Epidemiology (STROBE) guidelines. Ninety-three patients aged ≥60 years with LLD received an acute course of ECT and were stratified into mild-to-moderate anxiety (Hamilton Anxiety Rating Scale [HAMA] score 14–28) and severe anxiety (HAMA score ≥29) groups according to established HAMA severity cutoffs. Depressive symptoms (24-item Hamilton Depression Rating Scale [HAMD-24]), anxiety symptoms (HAMA-14), and cognitive performance (Montreal Cognitive Assessment [MoCA] and Repeatable Battery for the Assessment of Neuropsychological Status [RBANS]) were assessed at baseline and after completion of ECT treatment. Linear mixed-effects models (LMMs) with participant-level random intercepts were used to examine differential treatment trajectories while adjusting for demographic characteristics and baseline symptom severity. Hierarchical regression analyses were performed to determine whether improvement in anxiety symptoms (ΔHAMA) predicted antidepressant response (ΔHAMD) and cognitive change (ΔMoCA).

**Results:**

Both groups demonstrated significant reductions in depressive and anxiety symptoms following ECT. Baseline HAMA and HAMD-24 scores were not significantly correlated (*r* = 0.12, *p* = 0.24), suggesting that anxiety and depressive symptom severity represented distinct constructs. After multivariable adjustment, the severe anxiety group showed an antidepressant response comparable to that of the mild-to-moderate anxiety group (HAMD-24 total score interaction, *p* = 0.155), but exhibited significantly greater improvements in HAMA total and subdomain scores (all *p* < 0.01), as well as in the HAMD anxiety/somatization and retardation factors (both *p* < 0.05). Global cognitive performance remained stable across groups, with no significant differences observed in MoCA or RBANS total scores (both *p* > 0.60). Improvement in the RBANS attention domain was the only cognitive outcome that differed significantly between groups, favoring the severe anxiety group (*p* = 0.004). Greater improvement in anxiety symptoms predicted a stronger antidepressant response (*β* = 0.31, *p* < 0.001); however, this association did not differ between anxiety severity groups (*p* = 0.316) and was not related to cognitive change (*p* = 0.443).

**Conclusions:**

In patients with LLD, more severe baseline anxiety does not predict a greater overall antidepressant response to ECT but is associated with preferential improvement in anxiety-related and psychomotor symptom dimensions. ECT does not adversely affect global cognitive functioning regardless of baseline anxiety severity. These findings suggest that anxiety severity may help inform expectations regarding domain-specific symptomatic improvement and support the cognitive safety of ECT across the spectrum of anxiety severity in older adults.

**Clinical Trial Registration:**

This study is registered on the Chinese Clinical Trial Registry (ChiCTR) at https://www.chictr.org.cn/showproj.html?proj=205598 (registration number: ChiCTR2300078063).

## Main Points

1. Symptomatic Outcomes: After multivariate adjustment, severe baseline anxiety was associated with greater reduction in anxiety-specific symptoms (Hamilton Anxiety Rating Scale [HAMA] total and subdomains) and the Hamilton Depression Rating Scale [HAMD] anxiety/somatization and retardation factors following ECT compared with mild-to-moderate anxiety. However, overall antidepressant response, as indexed by HAMD-24 total score, did not differ significantly between subgroups.

2. Anxiety–Depression Association: Anxiety improvement was associated with antidepressant response, but was consistent across anxiety subgroups, indicating a uniform association rather than a moderating effect of baseline anxiety. Anxiety improvement was not associated with global cognitive change.

3. Cognitive Outcomes: Global cognitive performance remained stable across anxiety severity levels, and anxiety improvement was not associated with cognitive decline. Only the Repeatable Battery for the Assessment of Neuropsychological Status (RBANS) attention showed selective improvement in the severe anxiety group.

## 1. Introduction

Late-life depression (LLD) is a prevalent psychiatric disorder associated with high disability and significant social burden. Comorbid anxiety symptoms are common, affecting approximately 60%–70% of elderly patients with depression, and are associated with greater symptom burden, poor quality of life, and reduced rates of treatment response to pharmacotherapy [1,2]. Different levels of anxiety may represent distinct clinical and neurobiological subtypes of LLD that manifest through differences in dysfunction within the prefrontal-limbic circuit [3]. Studies have reported that LLD patients with high levels of anxiety exhibit greater resistance to antidepressant treatment [4,5,6], while also highlighting the effects of anxiety symptoms on disease prognosis [7,8], which may be related to the anxiety-associated overactivation of the hypothalamic-pituitary-adrenal (HPA) axis and the persistence of chronic inflammation [9,10]. Emerging evidence suggests that anxiety symptoms may reflect meaningful heterogeneity in depressive syndromes, potentially corresponding to distinct neurobiological and clinical profiles in affected patients [11]. Despite its clinical relevance, the severity of anxiety in LLD is often treated as a secondary feature rather than a stratifying dimension that may influence treatment selection and prognosis. Quantifying anxiety levels as a modifiable treatment factor may help realize precision and targeted treatments for LLD [3,12]. However, the practical implications of this heterogeneity remain insufficiently understood, and there is a pressing need to more fully explore the severity of anxiety symptoms in order to establish their relevance for stratification efforts aimed at facilitating personalized treatment.

Electroconvulsive therapy (ECT) is an effective treatment for LLD, especially in refractory cases, with an overall efficacy rate of 80%–90%. Existing studies suggest that high-anxiety LLD patients may exhibit unique neurobiological features, such as dysfunction in the limbic-cortical circuit together with HPA axis hyperactivation. ECT may specifically improve these abnormalities by regulating brain-derived neurotrophic factor (BDNF), serotonin (5-HT) systems, and prefrontal function [5,13]. Neuroimaging studies further indicate that high-anxiety patients exhibit unique electrophysiological susceptibility, with this subgroup showing differential ECT response profiles [5,14]. However, the practical implications of this heterogeneity among older adults receiving ECT remain insufficiently understood. There is a pressing need to more fully explore anxiety symptom severity to establish its relevance for risk stratification and personalized treatment allocation.

To the best of our knowledge, this is the first prospective study to stratify older adults with LLD according to their anxiety severity at baseline and to simultaneously examine both symptomatic and cognitive outcomes following bilateral ECT in this context. By treating the severity of anxiety as a stratification variable rather than a secondary feature, we provide initial evidence for differential symptomatic trajectories and cognitive safety profiles that may inform precision ECT treatment in geriatric psychiatry. This study was based on the hypotheses that: (1) ECT would significantly improve depressive and anxiety symptoms across different levels of anxiety severity; (2) Baseline anxiety severity would influence the magnitude of symptomatic improvement in anxiety-specific and psychomotor dimensions, though not necessarily overall depressive severity; and (3) Improved anxiety would be associated with antidepressant response or global cognitive change. By explicitly examining baseline anxiety severity and using it as a variable for patient stratification to explore differential ECT outcomes, the goal of this approach is to provide more precise and evidence-based recommendations for ECT-related clinical decision-making when managing LLD patients with anxiety symptoms.

## 2. Materials and Methods

This prospective self-controlled pre-post study was designed to evaluate the effect of ECT on depressive symptoms, anxiety symptoms, and cognitive function in LLD patients with varying levels of anxiety. The study was conducted in accordance with the Strengthening the Reporting of Observational Studies in Epidemiology (STROBE) guidelines.

### 2.1 Study Participants

Participants were recruited from elderly patients with LLD at Beijing Anding Hospital, between January 2024 and June 2025. Participants were required to meet the following inclusion criteria: (1) Age ≥60 years; (2) Diagnosis of major depressive disorder (MDD) according to the Diagnostic and Statistical Manual of Mental Disorders, Fifth Edition (DSM-5) criteria [15]; (3) Significant clinical anxiety symptoms, with a baseline Hamilton Anxiety Rating Scale (HAMA) total score ≥14 [16]; (4) Sufficient cognitive and literacy abilities to complete neuropsychological assessments (elementary school education or above); and (5) Clinically indicated for ECT. Exclusion criteria were as follows: (1) Significant cognitive impairment: Mini-Mental State Examination (MMSE) screening indicating cognitive deficits, or a Montreal Cognitive Assessment (MoCA) baseline total score ≤14 [17]; (2) Depression caused by other diseases, including but not limited to severe systemic diseases (e.g., severe cardiovascular, pulmonary, or immune disorders) and brain organic lesions; (3) History of head trauma, brain injury, epilepsy, or other neurological disorders; alcohol or substance abuse history; (4) The presence of devices such as implanted pacemakers, arterial clips, metal dental prosthetics, or bone fixation materials; and (5) Having undergone transcranial magnetic stimulation, transcranial direct current stimulation, or other physical therapies within the past 6 months.

Structured diagnostic interviews for comorbid anxiety disorders (e.g., generalized anxiety disorder [GAD], panic disorder) were not performed. Anxiety symptoms were operationalized dimensionally via 14-item Hamilton Anxiety Rating Scale (HAMA-14) scores rather than categorically via DSM-5 comorbidity. Patients were divided into two groups based on their baseline HAMA scores: a mild-to-moderate anxiety group (HAMA total score = 14–28), and a severe anxiety group (HAMA total score ≥29). These cutoffs were based on established HAMA-14 interpretation criteria originally developed by Hamilton (1959) [18] and subsequently validated in geriatric and clinical populations, with scores of 14–28 indicating mild-to-moderate anxiety, and scores ≥29 indicating severe anxiety. This threshold (≥29) has been widely adopted in Chinese clinical practice and prior geriatric studies to distinguish functionally impairing anxiety levels requiring intensive intervention [16,18].

### 2.2 ECT Treatment

ECT was administered 2–3 times per week, for a total of 6–12 sessions, depending on the patient’s clinical improvement and tolerance for the treatment. ECT was performed using a Spectrum 5000Q device (Mecta Corporation, Tualatin, OR, USA). Intravenous administration of atropine (0.5 mg), propofol (1–1.5 mg/kg), and succinylcholine (0.3–0.7 mg/kg) was used for anesthesia and muscle relaxation. Bilateral temporal electrode placement was performed, and the ECT settings included a wave width of 0.5 ms, with a frequency of 30 Hz. ECT dosage was determined using an age-based energy prediction method [19].

Short-term use of benzodiazepines was permitted (≤2 weeks) for patients with severe insomnia, with discontinuation being required at least 8 hours before each assessment. For anxiety or agitation, short-term use of lorazepam (≤3 mg/day) or oxazepam (≤45 mg/day) was permitted. Comorbid conditions were managed according to the standard of care and clinical guidelines, with efforts made to maintain stable medication types and dosages throughout the study. During the ECT course, no additional somatic treatments (e.g., transcranial magnetic stimulation, light therapy, electroacupuncture, biofeedback, vagus nerve stimulation) or structured psychotherapies (e.g., psychoanalysis, cognitive-behavioral therapy) were permitted, aside from general supportive therapy.

### 2.3 Assessment of Clinical Symptoms and Cognitive Function

This prospective cohort study assessed participants at two time points: baseline (within 24 hours before the first ECT session) and post-ECT (within 24–48 hours after the final ECT session). No intermediate or long-term follow-up assessments were performed. All 93 enrolled participants completed both assessments, and there was no loss to follow-up for the acute course outcomes reported herein.

The primary outcome was change in depressive symptom severity from baseline to post-ECT, as measured using the 24-item Hamilton Depression Rating Scale (HAMD-24) total score and operationalized as treatment response (≥50% reduction in HAMD-24 total score). Secondary outcomes included: (i) change in anxiety symptoms (ΔHAMA-14); (ii) change in global cognitive function (ΔMoCA total score); and (iii) change in domain-specific cognitive performance (ΔRepeatable Battery for the Assessment of Neuropsychological Status (RBANS) index scores).

#### 2.3.1 Assessment of Clinical Symptoms

A locally-designed questionnaire was administered to collect demographic information (e.g., age, gender, education) and clinical characteristics (e.g., age of onset, illness duration, medication history). Depressive symptom severity was assessed using the HAMD-24 [16]. A ≥50% post-treatment reduction in the total HAMD score was defined as an effective response. Anxiety symptom severity was assessed using the HAMA-14 [16]. The HAMD-24 and HAMA-14 scales were selected because they are widely validated in geriatric populations and capture somatic, anxiety, and cognitive symptom dimensions relevant to LLD. Scores of 14–28 (inclusive) indicated mild to moderate anxiety, whereas a score ≥29 indicated severe anxiety.

#### 2.3.2 Assessment of Cognitive Function

The MoCA was used to assess overall cognitive function, covering attention, executive function, memory, language, and visuospatial domains. A total score ≥26 indicated a normal level of cognition [20]. The RBANS tool was used to assess multiple cognitive domains through 12 subtests, including immediate memory, visuospatial construction, language, attention, and delayed memory. Higher scores in total and domain-specific subtests correspond to better cognitive function [21]. The RBANS was chosen for its sensitivity to transient cognitive changes and its established reliability in repeated assessments, making it suitable for evaluating ECT-related cognitive fluctuations.

### 2.4 Statistical Analysis

Data analysis was performed using IBM SPSS Statistics 26.0 (IBM Corp., Armonk, NY, USA) and Python 3.11 (Python Software Foundation, Wilmington, DE, USA; statsmodels 0.14). Distribution normality was assessed using the Shapiro-Wilk test. Continuous variables were summarized as mean ± standard deviation (SD) for normally distributed data or median (interquartile range, IQR) for non-normally distributed data. Categorical variables were reported as frequency (percentage) [n (%)]. Baseline demographic and clinical characteristics were compared between groups using the Mann-Whitney U tests for continuous variables, and chi-square tests or Fisher’s exact tests for categorical variables. Pearson correlations among HAMD-24, HAMA-14, MoCA, and RBANS scores at baseline were assessed to examine inter-scale associations.

#### 2.4.1 Differential ECT Response

To examine whether baseline anxiety severity predicted differential symptomatic and cognitive trajectories, linear mixed-effects models (LMMs) were fitted with restricted maximum likelihood (REML). Each participant was specified as a random intercept to account for within-subject correlation between pre- and post-ECT assessments. Fixed effects included time (pre-ECT vs. post-ECT), baseline anxiety subgroup (severe vs. mild-to-moderate), their two-way interaction, and covariates (age, sex, education years and baseline HAMD-24 severity). The time × group interaction was the primary parameter of interest, indicating whether the magnitude of change differed between subgroups. For HAMD-24 total score analysis, baseline HAMD-24 was omitted from covariates due to perfect collinearity with the pre-ECT outcome value in the repeated-measures structure. For HAMA, MoCA, and RBANS models, baseline HAMD-24 was retained as an additional covariate. For non-normally distributed outcomes, the robustness of REML estimation was confirmed via sensitivity analyses using log-transformed dependent variables; conclusions were materially unchanged.

#### 2.4.2 Within-Group Comparisons

Pre- and post-ECT score comparisons within each anxiety group were performed using Wilcoxon signed-rank tests for non-normally distributed symptom dimensions and paired-samples *t*-tests for cognitive scores where normality assumptions were met. MoCA total score change approximated normality and is presented as mean ± SD with paired *t*-tests, whereas RBANS subdomain changes are presented as median (IQR) with Wilcoxon signed-rank tests.

#### 2.4.3 Association Between Anxiety Improvement and Outcomes

Sequential (blockwise) multiple linear regression analysis was used to examine whether improvement in anxiety symptoms (ΔHAMA) was associated with antidepressant response (ΔHAMD) and cognitive change (ΔMoCA). Model 1 included baseline scores and anxiety subgroup as covariates; Model 2 additionally incorporated ΔHAMA; Model 3 further incorporated the ΔHAMA × anxiety subgroup interaction to test for differential associations across severity strata.

No formal a priori sample size calculation was performed for this exploratory study. Given the exploratory nature of this study and its small sample size, no formal adjustment for multiple comparisons was applied, and *p*-values should therefore be interpreted with caution. Missing data were minimal (<5% across all variables; HAMD-24 (0%), HAMA-14 (0%), MoCA (2.2%, n = 2), and RBANS attention (3.2%, n = 3)) and handled via complete-case analysis. The significance level was set at *p* < 0.05 (two-tailed).

## 3. Results

### 3.1 Demographic and Clinical Characteristics

A total of 93 patients with LLD met the inclusion criteria and were enrolled in this study. Based on baseline HAMA scores, participants were stratified into the mild-to-moderate anxiety group (n = 48) and the severe anxiety group (n = 45). The two groups did not differ significantly in demographic or clinical characteristics, including age, sex, marriage, years of education, age at onset, illness duration, family history of psychiatric disorders, ECT treatment, or Fluoxetine-equivalent doses (All *p* > 0.05). For further details, see Table 1.

**Table 1.** **Comparison of baseline characteristics and clinical features between patients with mild-to-moderate and severe anxiety**.

		Mild-to-moderate anxiety group	Severe anxiety group	*t*/*χ*^2^/*Z*	*p*
		(N = 48)	(N = 45)		
Age (years)		67.4 ± 4.0	66.7 ± 4.7	0.759	0.450
Sex (n, %)				0.194	0.660
	Male	15 (31.3)	16 (35.6)		
	Female	33 (68.8)	29 (64.4)		
Marriage (n, %)				1.060	0.303
	Married	39 (81.3)	40 (88.9)		
	Others	9 (18.8)	5 (11.1)		
Having a history of smoking (n, %)		9 (18.8)	9 (20.0)	0.023	0.879
Having a history of drinking alcohol (n, %)		5 (10.4)	5 (11.1)	0.012	0.914
Education [years, M (P_25_, P_75_)]		9 (9, 12)	12 (9, 12)	–1.217	0.224
Age at onset [years, M (P_25_, P_75_)]		58 (47, 66)	60 (51, 65)	–0.069	0.945
Course of the disease [months, M (P_25_, P_75_)]		96 (24, 234)	60 (24, 234)	–0.570	0.569
Number of previous depressive episodes [M (P_25_, P_75_)]		2 (1, 3)	3 (2, 4)	–1.498	0.134
Having precipitating factors (n, %)		29 (60.4)	28 (62.2)	0.032	0.858
Having a family history of psychiatric disorders (n, %)		19 (39.6)	18 (40.0)	0.002	0.967
Average number of ECT episodes [M (P_25_, P_75_)]		7 (6, 8)	8 (6, 9)	–1.855	0.064
Fluoxetine-equivalent doses [mg, M (P_25_, P_75_)]		23.6 (20.0, 45.1)	40.0 (17.5, 52.6)	–1.197	0.709

Note: M (P_25_, P_75_) represents the median (25th percentile, 75th percentile). ECT, electroconvulsive therapy.

### 3.2 Baseline Correlations Among Rating Scales

At baseline, HAMA total scores were not significantly correlated with HAMD total scores (*r* = 0.12, 95% CI: −0.08 to 0.32, *p* = 0.24; Table 2), indicating that anxiety and depressive severity represented largely distinct constructs in this sample. HAMD total scores showed a moderate negative correlation with MoCA total scores (*r* = −0.32, 95% CI: −0.50 to −0.13, *p* = 0.002; Table 3), suggesting that greater baseline depression was associated with poorer global cognition. HAMA total scores were not significantly correlated with MoCA total scores (*r* = −0.01, *p* = 0.93) or RBANS total scores (*r* = 0.18, 95% CI: −0.04 to 0.38, *p* = 0.10) at baseline (Table 3), indicating that baseline anxiety severity was not merely a proxy for overall cognitive impairment.

**Table 2.** **Comparison of HAMA and HAMD scores before and after ECT between the two anxiety-based groups, with multivariate-adjusted between-group differences in change scores**.

Variable	Group	Before ECT	After ECT	Within–group	LMM	
		Median (IQR)	Median (IQR)	*Z*/*p*	*β* (95% CI)	*p*
Total score of HAMD-24	Mild-to-moderate group	35.00 (31.00, 42.00)	14.00 (11.25, 16.00)	–6.034/<0.001*	−2.33 (−5.53 to 0.88)	0.155
	Severe group	38.00 (33.00, 41.50)	12.50 (11.00, 15.00)	–5.779/<0.001*		
Anxiety/somatization factor	Mild-to-moderate group	11.00 (10.00, 12.75)	6.00 (5.00, 8.00)	–5.918/<0.001*	−1.17 (−2.16 to −0.17)	0.022*
	Severe group	11.00 (10.00, 13.00)	5.00 (4.00, 6.00)	–5.803/<0.001*		
Cognitive disturbance factor	Mild-to-moderate group	4.00 (2.00, 6.75)	1.00 (0.00, 1.00)	–5.789/<0.001*	−0.77 (−1.51 to −0.03)	0.786*
	Severe group	5.00 (3.00, 7.00)	1.00 (0.00, 1.00)	–5.534/<0.001*		
Weight loss factor	Mild-to-moderate group	1.00 (1.00, 2.00)	0.00 (0.00, 0.00)	–5.103/<0.001*	−0.45 (−1.32 to 0.42)	0.312
	Severe group	1.00 (0.00, 2.00)	0.00 (0.00, 0.00)	–4.844/<0.001*		
Retardation factor	Mild-to-moderate group	7.00 (6.00, 8.75)	4.00 (3.00, 4.00)	–5.993/<0.001*	−0.05 (−0.41 to 0.31)	0.042
	Severe group	7.00 (6.00, 8.00)	3.00 (2.00, 4.00)	–5.779/<0.001*		
Sleep disturbance factor	Mild-to-moderate group	6.00 (4.00, 7.00)	1.00 (0.00, 2.00)	–5.795/<0.001*	−0.25 (−1.35 to 0.86)	0.312
	Severe group	7.00 (6.00, 8.00)	2.00 (1.00, 3.00)	–5.749/<0.001*		
Diurnal variation	Mild-to-moderate group	0.00 (0.00, 0.75)	0.00 (0.00, 0.00)	–3.071/0.002*	0.22 (−0.06 to 0.49)	0.122
	Severe group	0.00 (0.00, 0.00)	0.00 (0.00, 0.00)	–2.005/0.045*		
Hopelessness	Mild-to-moderate group	5.50 (4.00, 6.00)	1.00 (0.00, 2.00)	–5.961/<0.001*	−0.08 (−0.82 to 0.66)	0.831
	Severe group	5.00 (4.00, 6.00)	2.00 (1.00, 2.00)	–5.743/<0.001*		
Total score of HAMA	Mild-to-moderate group	25.00 (23.00, 27.00)	13.00 (10.00, 15.00)	–6.036/<0.001*	−7.02 (−8.71 to −5.33)	<0.001*
	Severe group	31.00 (30.00, 34.00)	13.00 (11.00, 15.00)	–5.781/<0.001*		
Psychic anxiety factor	Mild-to-moderate group	14.00 (11.00, 16.00)	8.50 (7.25, 10.00)	–5.858/<0.001*	−4.93 (−6.23 to −3.63)	<0.001*
	Severe group	19.00 (18.00, 19.00)	8.50 (7.00, 10.00)	–5.792/<0.001*		
Somatic anxiety factor	Mild-to-moderate group	11.00 (9.00, 13.75)	4.00 (2.00, 6.00)	–6.041/<0.001*	−2.09 (−3.39 to −0.79)	0.002*
	Severe group	13.00 (12.00, 15.50)	4.00 (4.00, 6.00)	–5.722/<0.001*		

Note: HAMD-24, 24-item Hamilton Depression Rating Scale; HAMA-14, 14-item Hamilton Anxiety Rating Scale; IQR, interquartile range. Data are presented as median (IQR). Within-group comparisons were performed using Wilcoxon signed-rank tests. Between-group differential change: linear mixed model (LMM) time × group interaction, adjusted for age, sex, education years, and baseline HAMD-24 (except HAMD-24 total, where baseline HAMD was omitted due to perfect collinearity). *p < 0.05.

**Table 3.** **Comparison of MoCA and RBANS scores between the two anxiety-based groups before and after ECT, with multivariate-adjusted between-group differences in change scores**.

Variable	Group	Before ECT	After ECT	Within–group	LMM	
		Median (IQR)	Median (IQR)	*t*/*Z*/*p*	*β* (95% CI)	*p*
Total score of MoCA	Mild-to-moderate group	18.03 ± 5.35	18.41 ± 5.02	–0.554/0.582	−0.50 (−2.55 to 1.55)	0.632
	Severe group	18.69 ± 4.42	18.47 ± 5.80	0.450/0.655		
Visuospatial/Executive functions	Mild-to-moderate group	2.50 (1.25, 4.00)	3.00 (1.00, 4.00)	–0.206/0.837	0.49 (−0.10 to 1.08)	0.103
	Severe group	3.00 (1.00, 4.00)	3.00 (2.00, 4.00)	–1.595/0.111		
Naming	Mild-to-moderate group	3.00 (2.00, 3.00)	3.00 (2.00, 3.00)	–0.905/0.366	−0.07 (−0.32 to 0.18)	0.583
	Severe group	3.00 (2.00, 3.00)	3.00 (2.00, 3.00)	–0.504/0.614		
Attention	Mild-to-moderate group	5.00 (3.00, 6.00)	5.00 (3.00, 6.00)	–0.128/0.899	−0.21 (−0.93 to 0.51)	0.566
	Severe group	5.00 (4.00, 6.00)	5.00 (3.00, 6.00)	–0.894/0.371		
Language	Mild-to-moderate group	2.00 (1.00, 2.75)	2.00 (1.00, 2.00)	–0.961/0.337	−0.10 (−0.54 to 0.34)	0.643
	Severe group	2.00 (1.00, 3.00)	2.00 (1.00, 3.00)	–0.342/0.732		
Abstraction	Mild-to-moderate group	1.00 (1.00, 2.00)	1.00 (1.00, 2.00)	–0.577/0.564	−0.16 (−0.50 to 0.17)	0.346
	Severe group	1.00 (1.00, 2.00)	1.00 (1.00, 2.00)	–0.955/0.340		
Delayed recall	Mild-to-moderate group	0.00 (0.00, 1.75)	1.00 (0.00, 2.00)	–1.202/0.229	−0.25 (−0.79 to 0.28)	0.353
	Severe group	0.00 (0.00, 1.00)	0.00 (0.00, 2.00)	–0.155/0.877		
Orientation	Mild-to-moderate group	5.00 (4.00, 6.00)	5.00 (4.00, 6.00)	–1.627/0.104	−0.20 (−0.78 to 0.37)	0.484
	Severe group	5.00 (4.00, 6.00)	5.00 (3.00, 6.00)	–1.731/0.084		
Total score of RBANS	Mild-to-moderate group	101.50 (86.25, 126.25)	101.00 (83.00, 118.00)	–0.210/0.833	1.08 (−7.98 to 10.14)	0.815
	Severe group	106.00 (92.00, 126.50)	101.00 (91.00, 128.00)	–0.430/0.667		
Immediate memory	Mild-to-moderate group	85.00 (26.75, 100.00)	88.00 (25.00, 104.00)	–0.789/0.430	0.32 (−3.86 to 4.51)	0.880
	Severe group	31.00 (22.50, 93.00)	75.00 (21.00, 96.00)	–1.050/0.294		
Visuospatial/Constructional	Mild-to-moderate group	94.50 (38.25, 107.50)	96.00 (39.00, 110.00)	–0.508/0.611	−0.31 (−4.75 to 4.13)	0.891
	Severe group	44.00 (27.00, 104.50)	88.00 (23.00, 106.00)	–0.883/0.377		
Language	Mild-to-moderate group	84.00 (47.50, 95.75)	84.00 (49.00, 98.00)	–0.159/0.874	−0.09 (−4.46 to 4.29)	0.969
	Severe group	45.00 (32.00, 99.00)	81.00 (29.00, 95.00)	–0.675/0.500		
Attention	Mild-to-moderate group	96.50 (30.75, 114.75)	95.00 (35.00, 108.00)	–1.911/0.056	4.93 (1.60 to 8.26)	0.004*
	Severe group	36.00 (16.50, 106.00)	90.00 (25.00, 112.00)	–2.005/0.045*		
Delayed memory	Mild-to-moderate group	72.50 (33.25, 96.75)	75.00 (34.00, 96.00)	–1.431/0.152	−1.43 (−5.99 to 3.14)	0.540
	Severe group	39.00 (29.50, 82.50)	64.00 (32.00, 86.00)	–1.911/0.056		

Note: MoCA, The Montreal Cognitive Assessment; RBANS, The Repeatable Battery for the Assessment of Neuropsychological Status. Data are presented as median (IQR) or mean ± standard deviation. Within-group comparisons were performed using Wilcoxon signed-rank tests, except for MoCA total score (paired *t*-test). LMM: time × group interaction adjusted for age, sex, education, and baseline HAMD-24. *p < 0.05.

### 3.3 Comparison of HAMA and HAMD Scores Between Groups

At baseline, total HAMD scores and the majority of HAMD factor scores did not differ significantly between the two groups (all *p* > 0.05; Table 2), with the only exception being the HAMD sleep disturbance score, which was significantly higher in the severe anxiety group (*p* < 0.05; Table 2), and HAMA total and subdomain scores, which were higher by design. Following ECT, both groups exhibited significant reductions in total HAMD and HAMA scores, as well as all subscale scores (all *p* < 0.05; Table 2). Post‑ECT comparisons revealed that the severe anxiety group scored significantly lower than the mild‑to‑moderate group on the HAMD anxiety/somatization and retardation factors, but continued to score significantly higher on the sleep disturbance factor (all *p* < 0.05).

After adjusting for age, sex, education years, and baseline HAMD-24 severity, linear mixed-effects models revealed that the time × group interaction for HAMD-24 total score did not reach significance (*β* = −2.33, 95% CI: −5.53 to 0.88, *p* = 0.155; Table 2), indicating comparable overall antidepressant response across anxiety subgroups. However, significant differential improvement favoring the severe anxiety group was observed for the anxiety/somatization factor (*β* = −1.17, 95% CI: −2.16 to −0.17, *p* = 0.022; Table 2) and the retardation factor (*β* = −0.77, 95% CI: −1.51 to −0.03, *p* = 0.042; Table 2). No significant differential associations were observed for cognitive disturbance, weight loss, sleep disturbance, hopelessness, or diurnal variation (all *p* > 0.05; Table 2).

For anxiety-specific outcomes, severe baseline anxiety was associated with greater reduction in HAMA total score (*β* = −7.02, 95% CI: −8.71 to −5.33, *p* < 0.001; Table 2), psychic anxiety factor (*β* = −4.93, 95% CI: −6.23 to −3.63, *p* < 0.001; Table 2), and somatic anxiety factor (*β* = −2.09, 95% CI: −3.39 to −0.79, *p* = 0.002; Table 2).

Treatment response rates were similar between the groups at 89.6% (43/48) in the mild-to-moderate anxiety group and 93.3% (42/45) in the severe anxiety group (*p* > 0.05). Median HAMD reduction rate did not differ significantly between these groups [60.3% (IQR: 52.0–70.4) vs. 66.7% (IQR: 59.1–72.5), *p* > 0.05].

### 3.4 Comparison of MoCA and RBANS Scores

At both the baseline and post-ECT assessment time points, there were no significant differences in MoCA total scores or RBANS total or subscale scores between groups (all *p* > 0.05; Table 3). Within-group analyses revealed that only the severe anxiety group demonstrated a significant improvement in RBANS attention scores post-treatment (*p* = 0.045; Table 3).

After adjusting for age, sex, education years, and baseline severity, baseline anxiety severity was not associated with differential change in MoCA total score (*β* = −0.50, 95% CI: −2.55 to 1.55, *p* = 0.632; Table 3) or RBANS total score (*β* = 1.08, 95% CI: −7.98 to 10.14, *p* = 0.815; Table 3). Among MoCA subdomains, no significant adjusted associations were observed for visuospatial/executive functions, naming, attention, language, delayed recall, or orientation (all *p* > 0.05; Table 3). For the RBANS, only attention showed a significant association, with greater improvement in the severe anxiety group (*β* = 4.93, 95% CI: 1.60 to 8.26, *p* = 0.004; Table 3); all other RBANS indices showed no significant association (all *p* > 0.05; Table 3).

### 3.5 Association Between Anxiety Symptom Improvement and Clinical Outcomes

Regression analyses revealed that improvement in anxiety symptoms was associated with greater improvement in depressive symptoms after adjusting for baseline HAMD total score (see Table 4). In contrast, ΔHAMA was not associated with changes in global cognitive function; baseline MoCA total score was the only variable significantly associated with cognitive change (*β* = –0.40, *p* < 0.001; see Table 5). Critically, the interaction term (ΔHAMA × anxiety subgroup) was non-significant in both models (both *p* > 0.05), indicating that the association with anxiety improvement on either depressive or cognitive outcomes did not differ between patients with mild-to-moderate versus severe baseline anxiety, suggesting a consistent effect across anxiety levels.

**Table 4.** **Hierarchical regression analysis predicting improvement in depressive symptoms (ΔHAMD) based on anxiety symptom improvement (ΔHAMA)**.

Model and predictors		*B*	*SE*	*β*	*t*	*p*	95% CI
Model 1 (constant)		10.48	1.99		5.274	<0.001*	6.54, 14.42
	Baseline HAMD	–0.84	0.05	–0.83	–15.460	<0.001*	–0.95, –0.73
	Baseline sleep factor	–0.26	0.19	–0.08	–1.376	0.172	–0.64, 0.12
	Anxiety subgroup	–1.00	0.75	–0.07	–1.341	0.183	–2.48, 0.48
Model 2 (constant)		5.34	2.02		2.645	0.009*	1.34, 9.35
	Baseline HAMD	–0.82	0.05	–0.80	–16.840	<0.001*	–0.91, –0.72
	Baseline sleep factor	–0.29	0.17	–0.08	–1.720	0.088	–0.63, 0.04
	Anxiety subgroup	2.12	0.89	0.14	2.383	0.019*	0.36, 3.88
	△HAMA	0.42	0.08	0.31	5.262	<0.001*	0.26, 0.57
Model 3 (constant)		5.22	2.02		2.582	0.011*	1.21, 9.24
	Baseline HAMD	–0.81	0.05	–0.80	–16.538	<0.001*	–0.91, –0.71
	Baseline sleep factor	–0.31	0.17	–0.09	–1.789	0.077	–0.65, 0.03
	Anxiety subgroup	2.28	0.90	0.15	2.526	0.013*	0.49, 4.07
	△HAMA	0.19	0.24	0.14	0.765	0.446	–0.30, 0.67
	△HAMA × Anxiety subgroup	0.16	0.16	0.18	1.007	0.316	–0.16, 0.48

Note: ΔHAMA, change in HAMA score (post-treatment minus baseline), with higher values indicating greater anxiety symptom improvement; *B*, unstandardized regression coefficient; *SE*, standard error; *β* (Beta), standardized regression coefficient; CI, confidence interval. *p < 0.05.

**Table 5.** **Hierarchical regression analysis predicting change in global cognitive function (ΔMoCA) from anxiety symptom improvement (ΔHAMA)**.

Model and predictors		*B*	*SE*	*β*	*t*	*p*	95% CI
Model 1 (constant)		6.10	3.27		1.863	0.066	–0.40, 12.60
	Baseline MoCA total score	–0.38	0.10	–0.39	–3.914	<0.001*	–0.57, –0.19
	Baseline HAMD	0.05	0.07	0.09	0.777	0.439	–0.08, 0.19
	Baseline sleep factor	–0.05	0.23	–0.02	–0.212	0.833	–0.51, 0.41
	Anxiety subgroup	–0.56	0.91	–0.06	–0.614	0.541	–2.37, 1.25
Model 2 (constant)		5.41	3.40		1.594	0.114	–1.35, 12.17
	Baseline MoCA total score	–0.39	0.10	–0.40	–3.980	<0.001*	–0.59, –0.20
	Baseline HAMD	0.05	0.07	0.09	0.813	0.418	–0.08, 0.19
	Baseline sleep factor	–0.06	0.24	–0.03	–0.268	0.790	–0.53, 0.40
	Anxiety subgroup	0.09	1.25	0.01	0.073	0.942	–2.40, 2.58
	△HAMA	0.09	0.11	0.10	0.770	0.443	–0.14, 0.31
Model 3 (constant)		5.40	3.43		1.573	0.119	–1.42, 12.22
	Baseline MoCA total score	–0.39	0.10	–0.40	–3.929	<0.001*	–0.59, –0.19
	Baseline HAMD	0.06	0.07	0.09	0.808	0.421	–0.08, 0.19
	Baseline sleep factor	–0.06	0.24	–0.03	–0.268	0.789	–0.53, 0.41
	Anxiety subgroup	0.10	1.27	0.01	0.080	0.937	–2.42, 2.62
	△HAMA	0.07	0.35	0.08	0.198	0.843	–0.62, 0.76
	△HAMA × Anxiety subgroup	0.01	0.22	0.02	0.055	0.956	–0.43, 0.45

Note: ΔMoCA, change in MoCA score (post-treatment minus baseline), with higher values indicating greater cognitive improvement; ΔHAMA, change in HAMA score (post-treatment minus baseline), with higher values indicating greater anxiety symptom improvement. *p < 0.05.

To examine whether improvement in anxiety symptoms was associated with greater improvement in depressive symptoms after accounting for baseline severity, a regression analysis was conducted with ΔHAMD as the dependent variable (Table 4). Model 1 included baseline HAMD total score. Baseline HAMD sleep disturbance factor scores were included as a covariate in repeated-measures ANOVAs and sequential regression models because they differed significantly between anxiety groups at baseline (Table 2), and prior research indicates that baseline sleep disturbance is associated with ECT outcomes [4]. This was a post-hoc statistical adjustment decision based on observed baseline imbalance rather than an a priori covariate selection. This model was significant (R^2^ = 0.77, F (3, 102) = 113.6, *p* < 0.001). In Model 2, ΔHAMA was added, leading to a significant increase in explained variance (ΔR^2^ = 0.05, *p* < 0.001), yielding a final R^2^ = 0.82, F (4, 101) = 114.4, *p* < 0.001. Within this model, ΔHAMA was significant positively associated with ΔHAMD (B = 0.42, 95% CI: 0.26–0.57, *β* = 0.31, *t* = 5.262, *p* < 0.001), indicating that each one-point decrease in anxiety symptoms corresponded to a 0.417-point decrease in depressive symptoms. Baseline HAMD showed the strongest negative association (*β* = –0.80, *p* < 0.001). Finally, Model 3 additionally incorporated the ΔHAMA × anxiety subgroup interaction term, which did not significantly improve model fit (ΔR^2^ = 0.00, *p* = 0.316), and the interaction itself was non-significant (B = 0.16, *p* = 0.316), indicating that the relationship between ΔHAMA and ΔHAMD did not differ across anxiety subgroups.

A parallel hierarchical regression analysis was conducted examining whether improvement in anxiety symptoms (ΔHAMA) was associated with change in global cognition (ΔMoCA; Table 5). Model 1 included baseline MoCA, baseline HAMD, baseline HAMD sleep factor, and anxiety subgroup as covariates. While the model was statistically significant, it explained only limited variance (R^2^ = 0.18, F (4, 92) = 4.93, *p* = 0.001). Model 2 additionally incorporated ΔHAMA, but this did not lead to any significant increase in explanatory power (ΔR^2^ = 0.00, *p* = 0.443). Only baseline MoCA emerged as a significant negative predictor of ΔMoCA (B = –0.39, 95% CI: –0.59 to –0.19, *β* = –0.40, *p* < 0.001), reflecting regression to the mean. ΔHAMA was not significantly associated with ΔMoCA (B = 0.09, *β* = 0.109, *p* = 0.443). Finally, Model 3 incorporated the ΔHAMA × anxiety subgroup interaction, but this did not improve model fit (ΔR^2^ = 0.00, *p* = 0.956).

Collectively, these findings indicate that, while anxiety symptom improvement was robustly associated with greater antidepressant response, it was not associated with changes in global cognitive function following ECT. Critically, the interaction term (ΔHAMA × anxiety subgroup) was non-significant in both models. Therefore, these findings do not support statistical moderation. Instead, they indicate that greater baseline anxiety severity was associated with greater absolute symptomatic improvement, although improvements in anxiety were similarly associated with antidepressant response across groups.

## 4. Discussion

In this stratified analysis of older adults with LLD, we found that baseline anxiety severity was associated with differential symptomatic improvement following ECT, specifically along anxiety-related and psychomotor dimensions, without differential impact on global cognition. After adjusting for demographic confounders and baseline severity, severe baseline anxiety predicted significantly greater reduction in HAMA total and subdomain scores, as well as in the HAMD anxiety/somatization and retardation factors. However, overall antidepressant response—as indexed by HAMD-24 total score—did not differ significantly between subgroups, suggesting that baseline anxiety severity shapes ECT outcomes selectively rather than globally. Global cognitive performance remained stable across anxiety severity levels, and anxiety improvement was not associated with cognitive decline. These findings suggest that the severity of comorbid anxiety may represent a clinically meaningful stratification factor for domain-specific ECT outcomes in older adults, particularly in anticipating preferential relief of anxiety and psychomotor symptoms without compromising cognitive safety.

### 4.1 Impact of ECT on Depressive and Anxiety Symptoms

The weaker HAMA–HAMD correlation in our sample (*r* = 0.12, *p* = 0.24) likely reflects range restriction, as all participants met criteria for clinically significant anxiety (HAMA ≥14), truncating the lower end of anxiety severity. This absence of correlation supports our conceptualization of anxiety and depression as distinct, non-redundant constructs in this severely anxious LLD cohort [22]. Consequently, baseline anxiety severity may operate as a domain-specific stratification variable rather than a mere indicator of overall illness severity.

This study provides novel evidence that baseline anxiety severity is associated with differential symptomatic trajectories following ECT in LLD, though this effect appears selective to anxiety-related and psychomotor dimensions rather than global depressive severity. After adjusting for age, sex, education years, and baseline HAMD-24 severity, the time × group interaction for HAMD-24 total score did not reach significance (*p* = 0.155), indicating comparable overall antidepressant response across anxiety subgroups. However, severe baseline anxiety predicted significantly greater improvement in HAMA total and subdomain scores, as well as in the HAMD anxiety/somatization and retardation factors (Table 2). These findings indicate that ECT may exert preferential effects on anxiety and psychomotor symptoms in severely anxious LLD patients, even when global depressive severity improves to a similar degree.

In our study, patients in the severe anxiety group exhibited higher baseline HAMD sleep disturbance scores as compared with those in the mild-to-moderate anxiety group (Table 2), consistent with the established link between anxiety and insomnia. However, after multivariate adjustment, baseline anxiety severity was not significantly associated with differential sleep disturbance improvement (*p* = 0.312), indicating comparable sleep outcomes across subgroups when baseline severity is accounted for. The preferential improvement in somatic anxiety and psychomotor retardation likely reflects ECT’s modulation of limbic-prefrontal circuits [23] and HPA axis activity [4,5,24,25,26,27,28,29]. It is noteworthy that LLD with prominent anxiety may overlap with agitated or mixed affective states, which are more resistant to pharmacotherapy and respond robustly to ECT. Future studies should incorporate DSM-5 mixed features assessments to clarify whether the observed differential response is driven by mixed-state biology rather than anxiety severity per se.

### 4.2 Impact of ECT on Cognition

Our study did not detect significant cognitive impairment in either group at baseline, nor significant decline in RBANS or MoCA total scores after ECT. After multivariate adjustment, baseline anxiety severity was not associated with differential change in MoCA total score or RBANS total score (Table 3). Among cognitive subdomains, only RBANS attention showed a significant adjusted association with baseline anxiety subgroup, with greater improvement observed in the severe anxiety group (Table 3). This selective association suggests that the effects of ECT on specific cognitive domains, rather than global cognition, may vary according to baseline anxiety severity.

This finding aligns with recent evidence from Loef and colleagues [14], who found no significant MMSE change in 426 patients (*β* = 0.10, *p* = 0.58), supporting the overall cognitive safety of ECT. However, their study also detected domain-specific effects—namely, deterioration in verbal learning alongside improved visual learning and motor speed—suggesting that ECT exerts selective rather than uniform cognitive effects. In our LLD sample, neither MoCA nor RBANS total scores declined; instead, RBANS attention improved selectively in the severe anxiety group. This discrepancy likely reflects two factors. First, Loef’s cohort had a mean age of 49 years, whereas our participants were all ≥60 years old. The MMSE used in their study as a global anchor has very low sensitivity (3.6%–11.1%) for detecting objective cognitive side effects such as verbal recall decline, whereas our RBANS and MoCA captured attention and executive function with greater granularity. Second, Loef’s study [14] did not examine anxiety severity as a predictor of cognitive outcomes. Our results indicate that baseline anxiety status does not confer additional cognitive risk in older adults and may even be associated with improved attention as emotional symptoms resolve.

This selective association likely reflects freed cognitive resources as emotional symptoms resolve, rather than superior global cognitive outcomes [30,31,32]. ECT may enhance prefrontal synaptic plasticity and inhibit default mode network overactivation [31], releasing cognitive capacity previously consumed by rumination and hyperarousal. Previous studies indicate that cognitive improvement after ECT often parallels reductions in depressive symptoms [33,34], possibly via BDNF-mediated hippocampal neurogenesis [35,36,37]. In our hierarchical regression analyses, however, anxiety symptom improvement (ΔHAMA) was not associated with change in global cognition (ΔMoCA), and the ΔHAMA × anxiety subgroup interaction was non-significant for cognitive outcomes (both *p* > 0.05), supporting a dissociation between ECT’s emotional and cognitive effects across the anxiety severity spectrum. All patients received bilateral ECT; future randomized studies should compare electrode placements (bilateral vs. right unilateral ultrabrief pulse) in anxious LLD patients to optimize the efficacy-cognition tradeoff [38].

### 4.3 Relationship Between Improvement in Anxiety Symptoms, Antidepressant Efficacy, and Cognition

The present results highlight an important pattern of symptom improvement after ECT treatment. Specifically, improvement in anxiety symptoms was significantly associated with antidepressant efficacy, but was not significantly associated with global cognitive function. Critically, the relationship between anxiety improvement and antidepressant response did not differ across baseline anxiety severity levels (ΔHAMA × subgroup interaction: *p* = 0.316), indicating a consistent association rather than a moderating effect of baseline anxiety.

These findings suggest that improvement in anxiety and depressive symptoms during acute ECT treatment may arise from shared neurobiological pathways [5,13]. Neuroimaging studies have shown that the antidepressant effects of ECT are closely related to its modulation of the limbic system–prefrontal cortex circuit, particularly in regions such as the amygdala, hippocampus, and anterior cingulate cortex [14]. These regions are also key hubs for the generation of anxiety-related emotions. As such, ECT may produce a synergistic effect on both depression and anxiety by rapidly modulating this shared emotional circuit, such that their improvement is strongly correlated in clinical practice. However, this association reflects observational co-occurrence only; the hierarchical regression models do not establish that anxiety reduction causally drives antidepressant response.

In contrast, anxiety improvement was not associated with global cognitive change, and the ΔHAMA × anxiety subgroup interaction was likewise non-significant for cognitive outcomes (*p* = 0.956), indicating that these domains are mediated by different neural pathways across the anxiety severity spectrum [39,40]. Baseline MoCA total score was the only significant predictor of post-ECT cognitive change, underscoring its prognostic value and reflecting regression to the mean [41].

### 4.4 Limitations and Future Directions

This study provides evidence for the value of considering baseline anxiety severity when anticipating ECT outcomes in LLD. While overall antidepressant response was comparable across anxiety subgroups, the preferential improvement in anxiety-specific and psychomotor dimensions in severely anxious patients suggests that baseline HAMA severity may help inform domain-specific expectations rather than stratify global efficacy. Patients with severe anxiety and prominent somatization or psychomotor retardation may derive particular symptomatic benefit from ECT, though this should not be interpreted as superior overall antidepressant efficacy.

Several limitations to this study should be acknowledged. First, this was a naturalistic, non-randomized study, and the effects of residual confounding factors cannot be excluded. The sample size was determined by the number of eligible patients treated during the study period. As a result, the relatively small sample may have limited our statistical power to detect moderation effects. In addition, we did not apply formal corrections for multiple comparisons (e.g., Bonferroni or false discovery rate [FDR]), potentially leading to a greater risk of Type I error. These findings should therefore be considered exploratory and require replication in larger samples. Differences in medication-related effects or medical comorbidity may have also had some influence on our study outcomes. Second, long-term follow-up data were not collected for the enrolled patients, so the durability of efficacy and cognitive improvement cannot be determined. Third, the absence of neuroimaging or other biomarkers limits the potential for any mechanistic inference regarding circuit-level modulation. Fourth, the adverse effects of ECT (e.g., headache, muscle pain, nausea, and subjective cognitive complaints) were not systematically monitored using standardized adverse event scales in this study. Given the vulnerability of the geriatric population to ECT-related side effects, this represents a significant limitation. Future studies should incorporate structured approaches to assessing adverse events in order to fully characterize the tolerability profile of ECT across anxiety severity strata. Fifth, all patients received bilateral ECT; therefore, we cannot determine whether the observed cognitive stability is specific to bilateral treatment or would generalize to right unilateral approaches, which may offer cognitive advantages in older adults [42]. In addition, between-group comparisons of raw change scores may have been confounded by regression to the mean, given the differing levels of severity at baseline. We therefore emphasize post-treatment absolute scores and within-group effect sizes in the primary interpretation. Structured diagnostic interviews for comorbid anxiety disorders (e.g., generalized anxiety disorder [GAD], panic disorder) were not performed. Anxiety symptoms were operationalized dimensionally based on HAMA-14 scores rather than categorically via DSM-5 comorbidity. This represents a limitation, as participants with primary anxiety disorders may represent a clinically distinct subgroup from those with anxiety symptoms secondary to depression.

Future studies should conduct long-term follow-up to verify the sustainability of therapeutic efficacy and utilize multimodal neuroimaging to elucidate the specific regulatory mechanisms of ECT on the anxiety-retardation and sleep-wake circuits. Additionally, the combination of ECT with sleep interventions should be explored to optimize the management of residual symptoms, and clinical and biological markers should be integrated to build predictive models for personalized, precise treatment. Randomized comparisons of electrode placement (bilateral vs. right unilateral ultrabrief pulse) in anxious LLD patients are also warranted to optimize the balance between antidepressant efficacy and cognitive safety.

## 5. Conclusions

In older adults with LLD, more severe baseline anxiety predicts greater absolute improvement in anxiety-specific and psychomotor dimensions following ECT, though not in overall depressive severity. This association reflects a difference in absolute symptomatic improvement rather than a moderating effect, as the relationship between anxiety improvement and antidepressant response was consistent across anxiety subgroups. ECT does not impair global cognition regardless of baseline anxiety severity, and anxiety improvement is not associated with cognitive change. These findings suggest that baseline anxiety severity may help inform domain-specific outcome expectations—specifically, anticipating preferential relief of anxiety and retardation symptoms—while supporting the cognitive safety of ECT across the anxiety severity spectrum in LLD.

## Data Availability

The datasets used and analyzed during the current study are available from the corresponding authors on reasonable request.
